# Basic Considerations for Data Pooling Strategy in Multi-Regional Clinical Trials (MRCTs)

**DOI:** 10.1007/s43441-025-00744-8

**Published:** 2025-01-25

**Authors:** Jiali Song, Chen Ji, Meng Chen, Jun Dong, Chao Zhu, Haiyan Wu, Wei Zhang, Kezhou Zhang, Bing Yu, Yun Wang, Hua Zhang, Fan Jia, Yan Hou

**Affiliations:** 1 Department of Biostatistics, School of Public Health, Peking University, Beijing, China; 2 Key Laboratory of Carcinogenesis and Translational Research (Ministry of Education), Department of Lymphoma, Peking University Cancer Hospital & Institute, Beijing, China; 3 Sanofi China, Beijing, China; 4 AstraZeneca, Shanghai, China; 5 Amgen Inc, Thousand Oaks, CA USA; 6 Lilly China Drug Development and Medical Affairs Center, Shanghai, China; 7 MSD R&D China, Beijing, China; 8 Boehringer Ingelheim China, Shanghai, China; 9 Novo Nordisk China, Beijing, China; 10 R&D-Based Pharmaceutical Association Committee (RDPAC), Beijing, China

**Keywords:** Pooling strategy, Effect modifier, Sample size allocation, ICH E17

## Abstract

The National Medical Products Administration of China has been implementing ICH E17, which describes the general principles for planning and designing of multi-regional clinical trials (MRCTs), yet there are several ambiguities in the execution and conduct remains in China or East Asia. In specific, pooling strategy, effect modifiers (EMs), statistical analysis, sample size allocation and their impact in alignment with global trial remains a challenge. In this paper, we explore on the criteria mentioned above under the context of China. EMs need to be determined and identified from intrinsic and extrinsic factors which might have the impact to the drug on specific populations. If no EMs are found, we use pooling by regions to understand whether differences across East Asian population exists, and whether pooling by East Asian is necessary. Statistical models used in the analysis are also listed to estimate the drug effect in pooled populations. In summary, this paper outlines the details of the MRCTs practices in China and provides better insights in practice both domestically and internationally for any future improvements.

## Introduction

The National Medical Products Administration of China (NMPA) has been implementing International Council for Harmonization of Technical Requirements for Pharmaceuticals for Human Use (ICH) E17, titled “General Principles for Planning and Design of Multi-Regional Clinical Trials [1] ”, after China joining the ICH as a full member. The full implementation of ICH E17 in China brings numerous advantages. First, the guideline enhances the international collaboration, which prompts China to participate in the globally simultaneous development of drugs in accordance to the unified standards. In addition, the implementation of the guideline accelerates the registration process and the marketing of innovative drugs. It allows Chinese patients to have timely access to the result of new drug development. Lastly, as a globally advantage, by opening up the market in China, the total cost of innovative drug are reduced by avoiding implementation of repetitive local or regional trials [2]. With prompt access to the data on Chinese population participating in MRCTs, China’s regulators can review clinical data and make regulatory decisions more efficiently.

It is challenging for both sponsors and regulators to consider the scientific principles, reliability, and the operability in the conduct of MRCTs, in particular the allocation of sample size in Chinese population. The challenge remains in satisfying both global and domestic standards: a small allocated sample size may not meet the local regulatory requirements of adequately evaluating efficacy and safety. Conversely, if the allocated sample size is too large, the enrollment Chinese subjects may delay the enrollment of the entire trial, which increase the operational difficulties in the consideration of China joining MRCTs. The planning and conduct of MRCTs is a complex issue. It is not recommended to focus only on a specific proportion of Chinese sample size. We need a comprehensive scientific assessment.

Pooling by regions or subpopulations is an important strategy in Asian countries. Pooling multiple regions or subpopulations satisfies local regulatory requirements and risk-benefit assessment. The implementation of pooling strategy also benefits the conduct of global MRCT, in that it may require fewer Chinese patients and facilitate the enrollment in China.

The concept of pooling strategy is presented in ICH E17 guideline [1]. There is a need to further discuss the implementation details because multiple stakeholders could have different understandings and sometimes have difficulties in reaching a consensus. In this paper, we aim to describe the general considerations for the pooling strategy application when China participates in MRCTs. The consensus on pooling strategy application are described in following narratives: determination of pooling strategy, possible EMs, and sample size considerations to illustrate the conduct in practice. By unifying the understanding across the industry, we aim to promote the application and implementation of E17 in China.

## Effect Modifiers in Pooling

As mentioned in Sect. 1, the key to determine the pooling strategy is to find the true EMs. EMs are broadly defined as the intrinsic and extrinsic factors that may affect the therapeutic effects of drug. Intrinsic factors are related to individual genetic, physiological and pathophysiological status, such as age, gender and anthropometrical characteristics, biomarker information and disease severity. Intrinsic factors should help to define the subpopulations in that it extrapolates between-region clinical data [3]. External factors are defined as the culture and environment that the person resides such as ethnicity, region, medical practice, and environmental factors, etc. ICH E5 guideline has listed various intrinsic and extrinsic factors [3]. In the early stage before phase II clinical trial studies are developed, EMs are generally hidden behind factors such as ethnicity and region. For example, subjects from one region may share similar weight distribution pattern, and body weight is a general manifestation of genetic profile, food habits, and a certain complication. Thus, geographic regions or regulatory regions, or more specifically, ethnicity-related information can be seen as a composite or alternative factor for many intrinsic and extrinsic factors mentioned above, though many residual confounding factors still remain. It is optimal when EMs are identified prior to the initiation of the pivotal MRCT.

Some factors with a high potential to have an impact on therapeutic effect can be pre-defined and then verified step-by-step based on the data obtained during each phase of the clinical development. The process can be generally divided into two steps: data collection and data evaluation. In the data collection step, we should try our best to harvest information from various sources to determine the potential candidates, including biomarkers or other factors beyond the intrinsic and extrinsic factors listed in ICH E5. We should consider not only prognostic factors of disease but also predictive factors of efficacy, which involves reviewing medical and scientific literature, regulatory guidelines, and other publicly available information to collect disease and genetic information [4]. Relevant medical databases, such as the WHO disease database and various registrational studies could be retrieved to collect epidemiology and historical data. Lastly, local healthcare professionals are a great source of consultation to inform local clinical practices and treatments. In the evaluation step, we want to evaluate the potential impact of intrinsic and extrinsic factors to investigational drugs using the data available. Consider testing whether these factors are EMs as the apriori hypotheses, we want to use the historical information or the concurrent data to test. This is often performed by designing and conducting clinical trials or via methods of modeling and extrapolation from historical data, such as early phase trials, exposure-response analysis in toxicology, PK/PD models and investigator-initiated trials. In some cases, the results might indicate the potential confounding factors after completion of the pivotal clinical trial, necessitating further evaluations and EM identifications through more analyses or even additional clinical trials.

## The Process to Determine the Pooling Strategy

ICH E17 presents seven basic principles for MRCTs, the fourth is on that the use of the pooling strategy may help in sample size allocation and the assessment of consistency across regions. In specific, pooled regions and pooled subpopulations are the two of the most common strategies, with their definitions from E17 guideline described as follows [1]:

- ***Pooled regions***: *“Pooling some geographical regions*,* countries or regulatory regions at the planning stage*,* if subjects in those regions are thought to be similar enough with respect to intrinsic and/or extrinsic factors relevant to the disease and/or drug under study.”*
- ***Pooled subpopulations***: *“Pooling a subset of the subjects from a particular region with similarly defined subsets from other regions whose members share one or more intrinsic or extrinsic factors important for the drug development programme at the planning stage. Pooled subpopulations are assumed as ethnicity-related subgroup particular important in the MRCT setting.”*

**Fig. 1:**
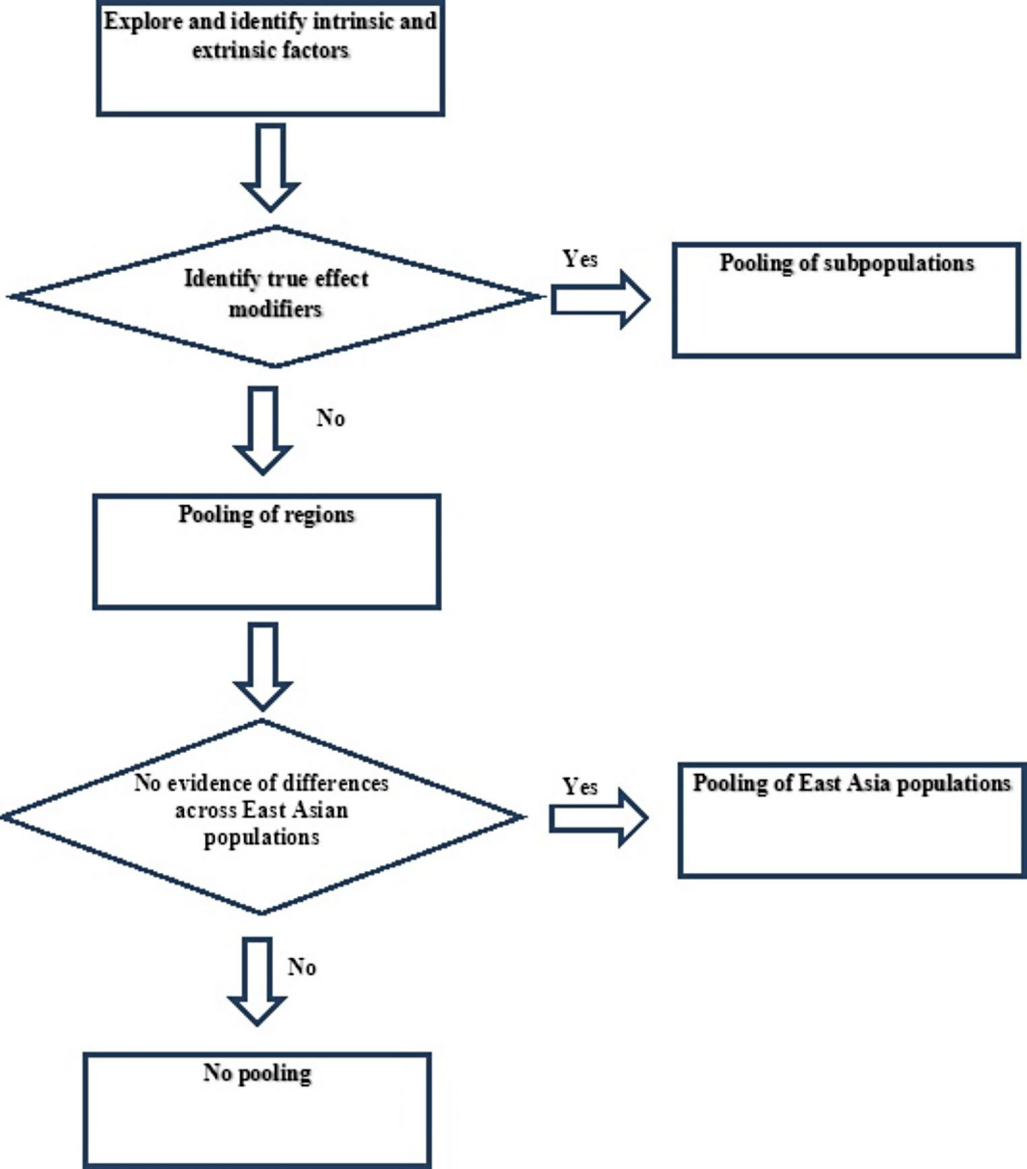
Process of determining the pooling strategy

The procedures to determine the pooling strategy in MRCTs are described in Fig. 1. First, we want to explore and identify the intrinsic and extrinsic factors that affect the therapeutic effects of drug or disease characteristics, which we define as the EMs. That is to say, the EMs should present differential treatment effect which has biological implications. Once the EMs are identified, we are able to define and pool different subpopulations according to the EMs. If the EMs cannot be clearly identified, a flexible pooling strategy on regions can be specified based on scientific arguments and practicability. If there are significant differences across regions, then no pooling should be conducted.

As mentioned above, we conduct data collection and data evaluation steps to determine the true EMs from the intrinsic and extrinsic factors. By identifying the true EMs, we are able to define pooled subpopulation by different levels of the EMs without discriminating on regions. In other words, stratification could be performed based on the level of EMs that impact the drug efficacy [4]. For example, in the PREVENT study, countries with similar distributions of effect modifiers were grouped into five pooled regions, and the clinical relevance and results were evaluated to identify potential effect modifiers [5, 6]. When more than one EM is taken consideration, the pooling strategies might be more complicated. In this case, cluster analysis can be used when there are multiple EMs [2]. The correlation between these EMs and endpoints can be used to define the distances between different populations. The populations can be pooled by using unsupervised learning methods such as hierarchical clustering and k-means clustering. However, it should be noted that the number of categories for clustering is generally no more than four [7, 8].

Pooling on the subpopulation by a specific attribute would yield the data that resembles a particular region, if this region also enriched with that attribute. This allows the pooled subpopulation to serve as a surrogate for evaluating the efficacy and safety within a specific region. For example, if a regional population has lower weight, a subpopulation that pools all low weight subjects from all regions can provide a good representation of the regional population. The subpopulation will support the assessment of drug efficacy in the regional population under the context of large sample size. Another practical example is if two populations share positive biomarker that solicit similar drug responses, then we could pool them together in the final analysis. If Chinese population makes up a high proportion of the biomarker positive group, then the other biomarker positive populations can be pooled with Chinese population together to help evaluate the drug efficacy in Chinese population.

However, it is extremely challenging to identify the true EMs. When the true EMs cannot be identified, we choose instead the region pooling strategy to assess the drug efficacy. We define the region pooling strategy using geographical location, country or regulatory region without discriminating on EMs. Often times, there are commonalities in terms of potential impact on efficacy and factor distribution in a specific region in for one or several specific EMs. When considering pooling of the entire Asian region versus the East Asian region, China has an inclination on pooling the East Asian population instead of the entire region Asian, due to similarity in EMs among east Asian countries.

Region pooling strategy allows adequate account of the potential etiology and eases on the operational practicability in trial conduct. Relevant data should be obtained from epidemiological and early clinical studies. In specific, ethnic sensitivity information may imply intrinsic factors such as pharmacokinetics and pharmacodynamics, genetic data, biomarkers, etc. Ethnicity is one of the most important information to identify the etiological differences in population by regions. In China, if no significant differences across different ethnicities are shown, the East Asian population can be pooled together. Or else, the region pooling strategy will no longer be considered, and we only focus on the Chinese population.

## Statistical Models for Evaluating Effect Size

The methods used for pooling approach depend on the number of EMs and the source of the population data that dictates the pooling method we use. We use regression model with an interaction term for one EMs, while multiple factors require multivariate models. The sources of pooled population data also imply data heterogeneity. If the pooled populations are from the same trial, the heterogeneity between populations is relatively small. If they are from multiple trials, between-trial differences and comparability across data sources should be considered. The most common approaches to address how to analyze data are described as follows. More statistical model details for analyzing the data can be found in reference [2].

**Simply pooling** is a direct pooling. There will be a generally descriptive summary on the baseline data, efficacy, safety and other endpoints for the pooled population. For the primary and key secondary endpoints, the pooled populations are considered as one subgroup and analyzed using the same model and approach as the overall study population.

**Fixed-effect model** assumes that the average treatment effects across the pooled populations come from the same distribution pooling, generally a weighted average approach is used for data integration and analysis.

**Random-effect model** assumes that there is a randomly various effect across populations for pooling, and generally the mixed-effects model is used for data integration and analysis.

**Data borrowing** provides an estimate for the current population by integrating external population data. In the same MRCT, if the number of subjects in Chinese population is limited, external data can be borrowed using the Bayesian methods [9]. Sources for external data may include different trials of the same drug, related trials of similar drugs, real-world data, etc. The key to data borrowing is to ensure the comparability between data from different sources, while also paying attention to the hidden confounding factors. Confounding factors can be eliminated by some methods as the propensity score matching.

## Sample Size Considerations After Pooling

There are five approaches for sample size allocation to regions listed in the ICH E17 guidelines, which encompasses the proportional allocation, equal allocation, preservation of effect, local significance, and fixed minimum number [1]. The sample size calculation should be considered for both the pooled populations and the Chinese population, which is debatable in the communication with China health authority.

In specific, five approaches for sample size allocation to regions listed in the ICH E17 guidelines are [1]:

- ***Proportional allocation***: *“Allocation of subjects to regions in proportion to size of region and disease prevalence.”*
- ***Equal allocation***: *“Allocation of equal numbers of subjects to each region.”*
- ***Preservation of effect***: *“Allocation of subjects to one or more regions based on preserving some specified proportion of the overall treatment effect.”*
- ***Local significance***: *“Allocation of a sufficient number of subjects to be able to achieve significant results within each region.”*
- ***Fixed minimum number***: *“Allocation of a fixed minimum number of subjects to a region.”*

The methods mentioned above each contains several notable drawbacks that should be considered carefully during implementation. The fixed minimum number/proportion approach is a convenient method, for example, allocating Chinese population to account for 10–20% of the overall study population. However, it might be too subjective and lack of scientific rationale. The local significance approach usually requires a larger sample size compared to other methods, resulting in a longer duration of enrollment. The preservation of effect approach often faces the challenges in defining a specific proportion of clinically meaningful treatment effects. The equal allocation approach often ignores the differences between regions.

## The Impact of Pooling Strategy on Drug Evaluation

Pooling strategy may cause confusion in that which analysis population should be mainly focused on. Regardless of any strategy being used, the primary study population to evaluate in an MRCT should be defined in the clinical trial protocol. Based upon the overall global study results, the pooling strategy focuses on the evaluation of the pooled population while also considering if it can represent Chinese population. It should be avoided that too few Chinese subjects are included in the pooled population.

The analysis of the primary population should be first considered under the context of a pivotal MRCT. For MRCTs in which China participates, when sponsors submit data to the regulator in China, an analysis of the Chinese population are often provided along with some supportive subgroup analyses by other confounding factors. The pooling strategy will prioritize the analysis of the pooled population before analyzing Chinese population and other subgroups (Table 1). In some cases, the pooled population is not completely including Chinese. The relation between the pooled population and Chinese population should be discussed.

**Table 1:** Impact of pooling strategy on the sequence of evaluating different populations

Sequence of population evaluation	Without pooling strategy	With pooling strategy
1	Primary analysis population	Primary analysis population
2	Chinese population	Pooled population
3	Related subpopulations	Chinese population and related subpopulations

## Concluding Comments and Discussions

This paper proposes specific ideas for the application of the pooling strategy in MRCTs based on the current practices in China. A flexible pooling strategy helps provide flexibility in sample-size allocation to China, facilitates the assessment of consistency in assessing the efficacy and safety of the drug. In addition, the relationship between the pooled population and the Chinese population should be discussed to ensure that the Chinese subjects are adequately represented. The conduct described in this paper aims to solidify the consensus between sponsors and regulators in understandings ICH E17, encourage China to participate in MRCTs, and accelerate the drug development and approval in China.

There are several key points that the industry should consider when communicating with the local regulatory authorities. When implementing the pooling strategy in MRCTs, it is recommended to communicate with the regulatory authorities as early as possible. This includes but not limited to the determination of the pooling strategy, the proper statistical analysis methods, and the sample size requirement. Documentation of the pooling strategy should be carefully stated. If the pooling strategy is used in a single MRCT, it should be clearly stated in the clinical study protocol and detailed in the statistical analysis plan. If a pooling strategy is used for multiple MRCTs, it should be documented in a separate file, such as the integrated statistical analysis plan.

During the entire process of clinical research and development, the pooling strategy may be adjusted based on early clinical trials results and the actual conduct of clinical research, either during the design stage or the conduct of the pivotal trial. Sponsors should discuss with regulatory authorities to clarify and document the final strategy prior to the locking of the study database. If sponsors and regulators cannot agree on the specific implementation of a pooling strategy, other approaches, such as the extension strategy, could be used to ensure an adequate number of Chinese patients are enrolled in the pivotal MRCT.

Our summary contains several limitations that are worth to consider in future attempts in practice. Pooling strategy can be extended from drug efficacy evaluation to safety evaluation. The EMs for efficacy measures are potentially different from those measuring the safety. In these cases, a different pooling strategy may be used. The pooling strategy applies not only to a single MRCT but also to multiple MRCTs. The latter is more complex in design and analysis, in which the confounding factors for trial differences need to be considered in pooling of the populations.

## Data Availability

No datasets were generated or analysed during the current study.
